# Potential therapeutic targets for chordoma: PI3K/AKT/TSC1/TSC2/mTOR pathway

**DOI:** 10.1038/sj.bjc.6605019

**Published:** 2009-04-28

**Authors:** N Presneau, A Shalaby, B Idowu, P Gikas, S R Cannon, I Gout, T Diss, R Tirabosco, A M Flanagan

**Affiliations:** 1 UCL Cancer Institute, University College London, 72 Huntley Street, London WC1E 6BT, UK; 2 Institute of Orthopaedics and Musculoskeletal Science, University College London, Stanmore, Middlesex HA7 4LP, UK; 3 Department of Histopathology, Royal National Orthopaedic Hospital NHS Trust, Stanmore, Middlesex HA7 4LP, UK; 4 The Sarcoma Unit, Royal National Orthopaedic Hospital NHS Trust, Stanmore, Middlesex, HA7 4LP, UK; 5 Department of Structural and Molecular Biology, University College London, Darwin Building, Gower Street, London WC1E 6BT, UK; 6 Department of Histopathology University College Hospital, University Street, London WC1E 6JJ, UK

**Keywords:** chordoma, mTOR, brachyury, AKT, rapamycin

## Abstract

Chordomas are radio- and chemo-resistant tumours and metastasise in as many as 40% of patients. The aim of this study was to identify potential molecular targets for the treatment of chordoma. In view of the reported association of chordoma and tuberous sclerosis complex syndrome, and the available therapeutic agents against molecules in the PI3K/AKT/TSC1/TSC2/mTOR pathway, a tissue microarray of 50 chordoma cases was analysed for expression of active molecules involved in this signalling pathway by immunohistochemistry and a selected number by western blot analysis. Chordomas were positive for p-AKT (92%), p-TSC2 (96%), p-mTOR (27%), total mTOR (75%), p-p70S6K (62%), p-RPS6 (22%), p-4E-BP1 (96%) and eIF-4E (98%). Phosphatase and tensin homologue deleted on chromosome 10 expression was lost in 16% of cases. Mutations failed to be identified in *PI3KCA* and *RHEB1* in the 23 cases for which genomic DNA was available. Fluorescence *in situ* hybridisation analysis for *mTOR* and *RPS6* loci showed that 11 of 33 and 21 of 44 tumours had loss of one copy of the respective genes, results which correlated with the loss of the relevant total proteins. Fluorescence *in situ* hybridisation analysis for loci containing *TSC1* and *TSC2* revealed that all cases analysed harboured two copies of the respective genes. On the basis of p-mTOR and or p-p70S6K expression there is evidence indicating that 65% of the chordomas studied may be responsive to mTOR inhibitors, rapamycin or its analogues, and that patients may benefit from combined therapy including drugs that inhibit AKT.

Chordoma is a rare malignant locally destructive tumour with a characteristic morphology and immunohistochemical profile (cytokeratin 19 and brachyury positive) that occurs, in the majority of cases, in vertebral bones and presents mostly with a large mass making wide excision rarely possible. Recurrent disease is a common event and metastases occur in as many as 40% of cases (Henderson *et al*, 2005; Romeo and Hogendoorn, 2006; Vujovic *et al*, 2006; Tirabosco *et al*, 2008; Hallor *et al*, 2008). Surgery, often ablative, is the main treatment option as the tumours are largely resistant to chemotherapy and radiotherapy, although proton/photon-beam radiotherapy is recommended for local control if available (Park *et al*, 2006) (for review see Casali *et al*, 2007).

Little is known about the genetic events that account for the development and progression of chordoma although there are reports that this tumour has developed in five patients with tuberous sclerosis complex. This is a tumour suppressor gene-related syndrome occurring on a background of germline mutations in *TSC1* and *TSC2* (encoding hamartin and tuberin, respectively). One of the cases harbouring a *TSC2* mutation had a clear loss of heterozygosity of the wild-type allele. The second case with a *TSC1* mutation revealed a reduced signal corresponding to the wild-type allele (allelic imbalance), and this was interpreted as loss of heterozygosity (Lee-Jones *et al*, 2004). Inactivation of TSC1/TSC2 function results in the phosphorylation of mTOR and its downstream effector molecules, the end result of which is initiation of translation, cell growth and proliferation (for review see Rowinsky (2004); MacKenzie and von Mehren (2007)). Inhibitors to mTOR are available and therefore the possibility exists of directed therapy for chordomas. mTOR exists in two molecular complexes, (mTOR complex mTORC1 and mTORC2), which exhibit different sensitivities to rapamycin and its analogues (Kim *et al*, 2002; Sarbassov *et al*, 2004; Yang *et al*, 2006b). However, investigation into mTOR activity in chordomas is required before offering such treatments as evidence also exists that the locus harbouring mTOR in chordomas is a region where there is frequent genomic loss (frequency of 0.57) (Scheil *et al*, 2001; Hallor *et al*, 2008).

The regulation of mTOR is complex, but in summary, AKT, a serine–threonine kinase, can be activated through a number of mechanisms, including the binding of a variety of growth factors to tyrosine kinase receptors, resulting in the phosphorylation of PI3K and PDK1 and subsequent inactivation of TSC1/TSC2 function, through phosphorylation of TSC2 at threonine 1462 by AKT. AKT can also be activated by RAS through PI3K, and through the assembled downstream TORC2 (Downward, 2004; Yang *et al*, 2006a), and activation of the RAS/MEK/ERK signalling cascade also induces tuberin phosphorylation independently of AKT (Roux *et al*, 2004). Inactivation of TSC2 releases a negative control on mTOR through the disinhibition of Rheb (RAS homologue enriched in brain), a small GTPase, and brings about phosphorylation of p70S6kinase (p70S6K) and 4E binding protein-1 (4E-BP1), and downstream phosphorylation of S6 ribosomal protein (RPS6) and eukaryotic translation initiation factor 4E (eIF-4E), respectively (Martin and Hall, 2005; Tee *et al*, 2005). Finally, activation of mTORC1 is also mediated by nutrients and branched amino acids, in particular (Wullschleger *et al*, 2006).

This study focuses on determining if there is evidence for the activation of PI3K/AKT/TSC1/TSC2/mTOR signalling in chordomas in view of existing therapeutic inhibitors.

## Materials and methods

### Clinical samples

This study complies with Central Office for Research Ethics Committees standards. The material, snap-frozen and paraffin-embedded tissue, was obtained from the histopathology department of The Royal National Orthopaedic Hospital. The clinical data were retrieved from the clinical notes. The cohort studied consisted of chordomas from 50 patients, 31 males and 19 females, with a median age of 65 years (range 19–84). The tumours were characterised by typical chordoma morphology and the co-expression of a pan-cytokeratin, cytokeratin 19 and brachyury. The sites of the tumours were sacrococygeal (*n*=41), lumbar (*n*=6) and cervical (*n*=3). In a period of follow-up ranging from 6 to 120 months, 46% of patients had one local recurrence, 12% had two and the remainder had no recurrences. Metastases involving bone, skin and lung occurred in 22% of patients. Frozen tumour was available from 23 primary tumours and paraffin embedded from all 50 cases (metastatic disease was not included). Five of the tumours had a dedifferentiated component but this material was only available from two cases and therefore the dedifferentiated component was not studied.

### Tissue microarray

The pathology was reviewed by AMF, AS and RT. A tissue microarray (TMA) of all 50 chordomas was constructed using a manual tissue arrayer (Beecher Instruments Inc., Sun Prairie, WI, USA). At least two representative 0.6 mm cores from each case were taken for the array. An additional 120 cores were used as control material and orientation markers and included human salivary gland, kidney, thyroid, tonsil, lymph node, placenta, testis, GIST, thyroid, renal cell and breast carcinomas.

### Antibodies and analysis of immunohistochemistry

Details of the antibodies used and conditions for the immunohistochemistry (IHC) are presented in Table 1. The IHC was performed either with the Ventana NexES Autostainer (Ventana Medical Systems, Strasbourg, France), Bond maX Immunostainer (Vision Biosystems, Newcastle upon Tyne, UK) or manually with ventana reagents. Diaminobenzidine was the chromogen in all reactions. Incubation without a primary antibody, as well as incubation with the matching IgG isotype control, under the same conditions for each antibody, served as negative controls.

Each antibody was scored with reference to a positive internal control on the TMA. The scoring system employed was as follows: negative in the absence of immunoreactivity, ‘low’ when the immunoreactivity was unequivocal but less strong than the positive control and ‘high’ when the immunoreactivity was at least as strong as the positive control. The positive cases showed immunoreactivity in more than 95% of the neoplastic cells. Non-neoplastic cells (macrophages, lymphocytes and fibroblasts) in the cores were used as internal negative controls.

### Protein isolation and western blot analysis

Ten 10 *μ*m sections were cut from snap-frozen resected specimens. Western blot analysis was carried out according to standard methodology. Briefly, protein extracts (30 *μ*g) were fractionated by SDS-8% polyacrylamide gel electrophoresis (unless otherwise stated) and transferred to a PVDF Immobilion-P transfer membrane (Millipore Corp., Bedford, MA, USA) by standard semi-dry electrotransfer methods. The membrane was blocked with blocking reagents (PBS, 0.1% Tween 20, 5% BSA) for a minimum of 30 min and probed with the appropriate primary antibody overnight at 4°C. Blots were washed in 1 × PBS with 0.1% Tween (PBS-T) and incubated for 1 h at room temperature with the appropriate secondary horseradish peroxidase-conjugated antibody, and enhanced using chemiluminescence detection (Amersham GE Healthcare, Buckinghamshire, UK).

The following antibodies were used for western blotting at a dilution of 1 : 1000 with overnight incubation at 4°C unless otherwise stated: p-AKT (ser 473) (dilution 1 : 250; Cell Signaling Technology, Danvers, MA, USA), total AKT (Cell Signaling Technology), isoform 1 and 2 of p70S6K, which were kindly provided by Professor Ivan Gout (University College London, UK), p-4E-BP1 (Thr 70) (Cell Signaling Technology), p-RPS6 (ser235/236) (Cell Signaling Technology), p-p70S6K (Cell Signaling Technology), S6 Ribosomal protein (clone 54D2; dilution Cell Signaling Technology), mTOR (clone 7C10; Cell Signaling Technology), GAPDH (Mab 6C5, dilution 1 : 5000, incubation 60 min at room temperature; Advanced Immunochemical Inc., Long Beach, CA, USA). Hela cells were used as a positive control when assessing for activation of PI3K/AKT/TSC1/TSC2/mTOR molecules (data not shown).

### Nucleic-acid isolation and mutational analysis by direct sequencing

Sampled neoplasms used for nuclei acid extraction comprised between 60 and 80% of tumour. PCR primers for each gene are described in Supplementary Table 1. DNA was extracted using proteinase K (Qiagen, Crawley, UK) from 23 frozen chordomas. Fifty to 100 ng of genomic DNA was amplified by the following touchdown PCR protocol, with annealing temperature reduced by 1°C per cycle from 65° to 56°C followed by 35 further cycles at 56°C as described above. The PCR was performed in a total volume of 50 *μ*l containing 5 *μ*l of 10 × Hotstart buffer I, 0.2 *μ*M of the dNTP mix, 100 pM of each primer, 1 U of Hotstart DNA polymerase (CLP, San Diego, CA, USA). PCR products were purified using Qiagen purification PCR kit (Qiagen) and DNA sequencing was performed at the Scientific Support Services, UCL. Sequencing reactions were run using GenomeLab DTCS Quick Start chemistry (Beckman Coulter (UK) Ltd Biomedical Research, Bucks, UK) and were analysed on a CEQTM8000 Genetic Analysis System (Beckman Coulter).

RNA was extracted from paraffin-embedded samples using Optimum FFPE RNA Isolation Kit (Ambion Europe, Huntington, Cambridgeshire, UK) and from frozen tissue using TRIzol reagent (Invitrogen Ltd, Paisley, Scotland, UK) followed by purification with RNeasy columns with an additional on-column DNAse I treatment (Qiagen) according to the manufacturer's instructions to avoid genomic DNA contamination. Approximately 150 ng of the total RNA extracted from each sample was reverse transcribed using Superscript III First-Strand Synthesis kit (Invitrogen) according to the manufacturer's instructions and using gene-specific primers for different exons to avoid amplification from gDNA (Supplementary Table 1).

### FISH analysis of *TSC1*, *TSC2*, *mTOR* and *RPS6*

Fluorescence *in situ* hybridisation (FISH) was performed on TMAs using probes from the RP11 BACs library. RP11-81C14, RP11-304L19, RP11-1107P2 and RP11-624N8 were used for assessing *TSC1, TSC2*, *mTOR* and *RPS6* allelic loss, respectively (BACPAC Resources Center, Oakland, CA, USA). Centromeric probes, CEP9 and CEP16, were used to assess the presence of two copies of chromosome 9 (*RPS6*) and 9 and 16 (*TSC1* and TSC2, respectively) (Vysis, Abbott Laboratories Inc., Des Plaines, IL, USA), and *α*-satellite D1Z5 was used for chromosome 1 (*mTOR*). The probes were prepared with reagents from Vysis. TMA sections were de-paraffinised in xylene and dehydrated in ethanol. Sections were pretreated using the Paraffin Pretreatment Reagent Kit II (Vysis): the slides were placed in the pretreatment solution at 80°C for 50–70 min and in pepsin solution 0.05% at 37°C for 20–25 min. The probe, applied to the slides, were co-denatured for 5 min at 73°C and hybridised for at least 16 h at 37°C in a Thermo-Brite hybridiser (Iris, Westwood, MA, USA). The slides were washed in formamide-free solutions: 2 × SCC/0.3% NP40 (Igepal) at room temperature for 5 min, at 73°C for 2 min and at room temperature for 1 min, and then counterstained with DAPI and analysed under a fluorescent microscope (Olympus, Watford, Hertfordshire, UK) using AnalySIS software (Olympus). The chromosome localisation of all BAC clones was confirmed on normal cells in metaphase. Fifty non-overlapping nuclei that contained unequivocal signals were counted for each case. Hemizygous deletion of *RPS6* and *mTOR* was defined as more than 20% of non-overlapping tumour nuclei containing one *RPS6* or *mTOR* locus red signal and by the presence of two CEP9 and D1Z5 green signals for *RPS6* and *mTOR*, respectively.

## Results

### Immunohistochemistry results

**Figure 1:**
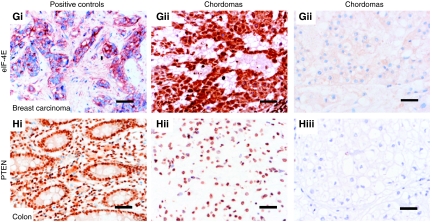
Transmitted light photomicrographs of chordoma characterised by cords and clusters of uniform cells with vacuolated cytoplasm set within a myxoid stroma (**Ai**) and immunoreactive (nuclear) for brachyury (**Aii**) and cytokeratin 19 (**Aiii**). The photomicrographs on the left-hand panel represent positive control tissues (breast carcinoma **Bi**, **Ci**, **Di**, **Ei**, **Fi**, **Gi**; colon **Hi**) that are immunoreactive for the indicated antibodies. The middle panel shows representative chordomas, which were scored as ‘strong’ for expression of the indicated antibody, indicating that the immunoreactivity is as strong as that in the positive control. The right-hand panel includes representative chordomas that were not immunoreactive for the relevant antibody. The bar=50 *μ*m.

More than 90% of the chordomas expressed p-AKT (Ser243), p-TSC2 (Thr1462), p-4E-BP1 (Thr70) and eIF-4E as assessed by IHC (Figure 1, Table 2). The labelling pattern of these different antibodies was largely similar and consisted of diffuse granular cytoplasmic immunoreactivity, although nuclear positivity for p-AKT and p-4E-BP1 was also noted in less than 10% of cases (Figure 1). In contrast, phosphorylation of mTOR (Ser2448), p70S6K (Thr389) and RPS6 (Ser235/236) was found in only 13 of 48 (27%), 29 of 46 (62%) and 11 of 49 (22%) cases, respectively (Table 2, Figure 1, and Supplementary Table 2).

### Correlation of immunohistochemistry and western blot results

**Figure 2:**
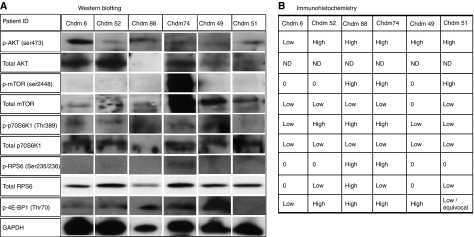
Validation of the immunohistochemistry results by western blotting for selected targets in the PI3K/AKT/TSC1/TSC2/mTOR pathway on six chordoma (Chdm) cases: (**A**) western blotting for p-AKT (ser473), total AKT, p-mTOR (ser2448), total mTOR, p-p70S6K (Thr389), isoform 1 of p70S6K, p-RPS6 (ser235/236), total RPS6 and p-4E-BP1 (Thr70). The membranes were stripped and reprobed with anti-GAPDH antibody to assure even loading of proteins in each lane. (**B**) IHC data for the samples analysed by western blot. Hela cells were used as a positive control when assessing for activation of PI3K/AKT/TSC1/TSC2/mTOR molecules (data not shown).

The western blot data on selected cases for p-AKT, p-4E-BP1 and p-p70S6K were in complete concordance with the IHC results (Figure 2). In addition to the phosphorylated status of p70S6K by IHC, all six chordomas analysed by western blot were positive for the rapamycin-sensitive S6K isoform 1, 70 kDa (Figure 2). Western blot analysis for p-mTOR confirmed the IHC data in four of six cases, showing the presence of the activated protein in one case and the absence in three cases.

The p-RPS6 IHC was confirmed in five of the six cases analysed by western blotting: the one discrepant result showed that one immunoreactive case was negative by western blot (Figure 2). Western blot analysis revealed total RPS6 protein in all these six cases although two of these were negative for total protein by IHC. These two cases revealed loss of one *RPS6* allele (*vide infra*), and both were negative for p-RPS6 by western blot, suggesting that the positive result for total RPS6 by western blot may be explained by non-neoplastic stromal cells contaminating the protein lysate.

### Correlation of *mTOR*-positive chordomas with other markers

Of the 13 (27%) immunoreactive chordomas for p-mTOR, 12 were immunoreactive for total mTOR protein and showed two copies of the *mTOR* by FISH, where data were available, and all showed phosphorylation of 4E-BP1 and expressed eIF-4E. Eleven of these 13 p-mTOR-positive cases showed activation of p-p70S6K, and 7 showed activation of p-RPS6. The two cases negative for p-p70S6K activation were also negative for p-RPS6 (Table 2 and Supplementary Table 2).

### Correlation of *mTOR*-negative chordomas with other markers

**Figure 3:**
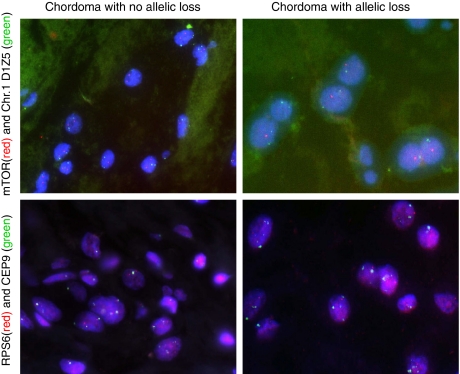
Photomicrographs of interphase fluorescent *in situ* hybridisation of chordomas showing an example of allelic loss (right-hand side columns) and the normal two copies (left-hand side columns) for *mTOR* (top row) and for *RPS6* (bottom row). The red signals identify *mTOR* and *RPS6*, respectively. The green signals represent chromosome 9 *α*-satellite probe CEP9 for *RPS6*, and the D1Z5 *α*-satellite probe identifies the chromosome 1 centromere for *mTOR*.

**Figure 4:**
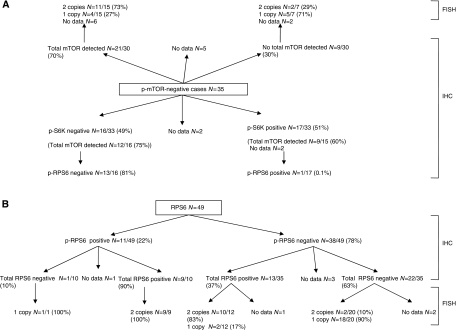
Schematic diagrams representing chordomas analysed for mTOR and p70S6K (**A**), and RPS6 (**A** and **B**) (total and activated forms) by immunohistochemistry (IHC) and fluorescent *in situ* hybridisation (FISH).

Thirty-five of 48 chordomas were negative for p-mTOR; 30 of these could be analysed and 21 (70%) were immunoreactive for total mTOR. As *mTOR* is found in a region reported to be frequently lost in chordomas and other neoplasms, the tumours were analysed for allelic loss by FISH (Figure 3). Of these 21 total mTOR-positive cases, 11 showed two *mTOR* alleles and 4 revealed loss of one allele (Figure 4A). This left 9 of 30 (30%) p-mTOR-negative cases exhibiting no total mTOR protein as assessed by IHC: five of these cases showed loss of one allele by FISH, two cases showed two copies of the gene and there were no data on two cases.

Approximately 50% of the p-mTOR-negative chordomas (16 of 33: 12 of which were immunoreactive for total mTOR) showed activation of neither p70S6K nor RPS6. The remaining 17 p-mTOR-negative chordomas were positive for p-p70S6K, 9 of 15 (60%) of which were immunoreactive for total mTOR, but only one of these cases was positive for RPS6 (Figure 4A).

### Correlation of RPS6-negative chordomas with other markers

Thirty-eight of 49 (78%) chordomas were negative for p-RPS6 and 22 of 35 analysable cases (no data for 3 cases of the 38) showed no expression of the total protein RPS6. Fluorescence *in situ* hybridisation data showed that 18 of the 20 (90%) analysable cases (two had no FISH data available out of the 22) had only one copy of the gene (Figure 4B). In total, 21 cases of 47 (47%) showed loss of one copy by FISH (Supplementary Table 2) (Figure 3).

In view of *RPS6* being located in the same chromosomal region as *CDKN2A* (*p16* (*INK4*)), a common tumour suppressor gene that is lost frequently in chordomas (Hallor *et al*, 2008), we speculated that the allelic loss of *RPS6* correlated with the loss of *CDKN2A*. This was confirmed by showing that CDKN2A was detected by IHC in only 5 of 48 chordomas (Table 2) and that these 5 cases showed no evidence of allelic loss for *RPS*6. Furthermore, total RPS6 protein was detected by IHC in four of these (no data for one case, Supplementary Table 2). In contrast, 10 CDKN2A-negative cases showed p-RPS6 positivity and 9 of these showed no *RPS6* allelic loss (no data for one case) (Supplementary Table 2).

### FISH results for *TSC1* and *TSC2*

Fluorescence *in situ* hybridisation for *TSC1* (28 of 28 cases) and *TSC2* (24 of 24 cases) showed two alleles.

### Phosphatase and tensin homologue deleted on chromosome 10 in chordoma

*Phosphatase and tensin homologue deleted on chromosome 10* (*PTEN*), a tumour suppressor gene, which when not expressed contributes to constitutive phosphorylation of AKT and activation of the downstream pathways, displayed no immunoreactivity in 16% of chordomas (Figure 1, Table 1). The absence of PTEN by IHC was confirmed by semi-quantitative RT-PCR (data not shown). The absence of PTEN expression did not correlate with the presence of metastatic disease, local recurrence or a dedifferentiated component.

### Mutations in *RHEB* and *PI3KCA* are not detected in chordomas

Direct sequencing for predicted mutations in *RHEB* codons 15, 16 and 64, which are similar to *K-RAS* codons 12, 13 and 61, respectively (Li *et al*, 2004; Yan *et al*, 2006), and for common mutations in *PI3KCA* (exons 4, 5, 7, 9 and 20), failed to reveal mutations (Samuels *et al*, 2004) (see Supplementary Table 3).

## Discussion

mTOR, a protein kinase, is a pivotal regulator of protein synthesis and controls entry into G1 phase of the cell cycle through phosphorylation of the substrates p70S6K and 4E-BP1, molecules that cooperate in translational initiation and ribosomal biogenesis. It is for these reasons that mTOR inhibitors are considered potential therapeutic agents in tumours where mTOR signalling is activated. On the basis of this study of 50 chordomas, we consider that there is evidence that 65% of the tumours are potentially responsive to mTOR antagonists. This figure is generated by the finding that 27% (*n*=13) of the chordomas exhibit phosphorylated mTOR and a further 18 cases express p-p70S6K although they are negative for p-mTOR.

p-p70S6K is generally used in preference to p-mTOR to assess the tumour response to mTOR antagonists and to predict which tumours may be responsive to such agents (for reviews see Boulay *et al* (2004); MacKenzie and von Mehren (2007)). Indeed, phosphorylated mTOR has been generally found to be expressed in relatively low numbers of tumours, and several studies make no reference to tumour mTOR expression when assessing the effect of rapamycin and or its analogues (Jaeschke *et al*, 2002; Gao *et al*, 2003; Krishnan *et al*, 2006; El-Salem *et al*, 2007; Lu *et al*, 2008; Scheper *et al*, 2008). Technical difficulties involved in detecting large phosphorylated proteins (mTOR 289 kDa) (Tkaczyk *et al*, 2002), in addition to the general transient expression of phosphorylated proteins, may contribute to the difficulty in detecting p-mTOR (Riemenschneider *et al*, 2006). mTOR phosphorylation may also occur on a variety of residues that we have not tested (Cheng *et al*, 2004).

We consider that the 9 (20%) chordomas that do not express mTOR (total and phosphorylated) protein as assessed by IHC (five of these nine revealed only one *mTOR* allele (two cases with both alleles, two cases no data)) are unlikely to respond to mTOR inhibitors. Furthermore, we also speculate that the 10 cases that express total mTOR but negative for p-mTOR, p-p70S6K and p-RPS6 would also be at high risk of not responding to mTOR antagonists. On the basis of this calculation, 35% of our chordomas would be unlikely to be responsive to mTOR antagonists.

The complete absence of mTOR expression in nine chordomas raises the question as to how cells survive in the absence of ribosomal biogenesis. One explanation is that the IHC for mTOR is not sufficiently sensitive or that very low levels of mTOR are required for assembling the protein complex (mTORC1). Alternatively, an mTOR-independent pathway, capable of bringing about ribosomal biogenesis and initiation of translation, may be activated. The report that RAS/MEK/ERK pathway activates RPS6 independently of mTOR through p90 ribosomal S6 kinase supports this postulate (Roux *et al*, 2004).

In view of the importance of ribosomal synthesis in cell growth, the absence of RPS6 protein expression in 58% of chordomas is striking but appears to be a robust result because the IHC data are supported by the finding of *RPS6* allelic loss in 21 of the 23 cases. Furthermore, the data correlate with the loss of *CDKN2A* that lies on chr 9p21.3 and is located adjacent to the cytoband harbouring *RPS6* (chr 9p22.1). Hence, the lack of expression of RPS6 protein expression suggests that other p70S6K substrates, such as splicing factor SCAR, translation initiation factor eIF-4B, EF-2B kinase and pro-apoptotic protein Bad1 (Harada *et al*, 2001; Raught *et al*, 2004; Richardson *et al*, 2004), which are less well studied, are likely to undertake this important function (Ruvinsky and Meyuhas, 2006).

It is not clear how loss of RPS6 contributes to the development of neoplasia although its allelic loss has also been reported in chondrosarcomas, a tumour with similarities to chordomas (Rozeman *et al*, 2006). Interestingly, a causal relationship between RPS6 phosphorylation and protein synthesis has revealed conflicting results. In addition to reports of activation of this protein resulting in elevated cell growth, there is also evidence of increased global protein synthesis in embryonic fibroblasts derived from rpS6^−/−^ mice compared with wild-type cells (Ruvinsky *et al*, 2005). Furthermore, germline mutations in *RPS19*, which occur in patients with Blackfan–Diamond syndrome, a cancer susceptibility syndrome associated with haematological malignancies, have already established a role for the loss of RP in cancer. Mutations in this syndrome are directly related to loss of expression of RPs, such as (RP)S19, RPL5 and RPL11 (Draptchinskaia *et al*, 1999; Cmejla *et al*, 2009).

One of the reasons for embarking on the analysis of the mTOR pathway in chordomas was the reported loss of heterozygosity in chordomas in two individuals with tuberous sclerosis complex syndrome (Lee-Jones *et al*, 2004). However, our finding that AKT is activated in over 90% of chordomas argues that the tumour suppressor genes, *TSC2* and *TSC1*, in sporadic chordoma are likely to be ‘inactivated’ not as a result of loss of heterozygosity but rather by phosphorylation of TSC2, at threonine 1462, by p-AKT (Inoki *et al*, 2002). Indeed, if the tumour suppressor genes were inactivated, the absence of the TSC2 protein would be expected and this was not the case.

The failure to detect *RHEB* and common *PI3K* mutations in 23 chordomas provides evidence that these genetic events are unlikely to account for chordoma tumourigenesis in most cases. Furthermore, the published array CGH data showing that neither *AKT* nor *PI3KCA* is amplified in 21 cases largely exclude activation of AKT through these genetic events (Hallor *et al*, 2008). However, these experiments are not exhaustive and additional analyses on greater numbers of genes, in larger tumour series, are warranted.

Functional studies are required to validate our findings, but accessing appropriate control material (notochord), the availability of only one slow-growing chordoma cell line, UCH-1 (Scheil *et al*, 2001), and the scarcity of animal models prevents this avenue of investigation (although we established xenografts from five different chordomas in nude mice, the tumours disappeared when passaged into a second animal (data not shown)), making this approach difficult. Nevertheless, our findings provide an evidence base for treating selected chordomas with mTOR, AKT inhibitors and antisense molecules to the attractive cancer target, eIF-4E (Graff *et al*, 2008). A combination of agents, rather than individual drugs, is more likely to offer therapeutic success particularly as there is evidence that AKT is activated at ser473 by TORC2, a rapamycin-insensitive molecule (Sarbassov *et al*, 2005). The development of a new generation of mTOR-blocking agents (TORKinibs) that interrupt the TORC2 complex is under development and may provide therapeutic benefit over what is currently available (Feldman *et al*, 2009).

## Figures and Tables

**Table 1:** Details of antibodies used for immunohistochemistry in this study

**Antibody/clone**	**Source**	**Antigen retrieval**	**Dilution**	**Incubation time and temperature**	**Method**
Cytokeratin 19 clone b170	Novocastra, Peterborough, UK	Protease, 20 min	1 : 100	30 min at 37°C	Ventana NexES autostainer
Brachyury	Santa Cruz, CA, USA	Pressure cooker, 2 min	1 : 50	30 min at 37°C	Ventana NexES autostainer
PTEN clone 28H6	Abcam, Cambridgeshire, UK	Pressure cooker, 2 min	1 : 100	30 min at 37°C	Ventana NexES autostainer
CINtec p16 ^INK4a^ clone E6H4TM	mtm laboratories AG, Heidelberg, Germany	Dako ERS, 40 min, Ely, Cambridgeshire, UK		20 min at 37°C	Bond maX immunostainer
p-AKT (ser 473) clone 736E11	Cell Signaling Technology, Danvers, MA, USA	Pressure cooker, 6 min	1 : 50	Overnight incubation at 4°C	Manually with Ventana reagents
Harmatin/TSC1	Cell Signaling Technology	Pressure cooker, 2 min	1 : 25	30 min at 37°C	Ventana NexES autostainer
Tuberin/TSC2	Santa Cruz	Pressure cooker, 2 min	1 : 100	30 min at 37°C	Ventana NexES autostainer
p-Tuberin/TSC2 (Thr1462) (clone ab59274	Abcam	Pressure cooker, 4 min	1 : 25	Overnight incubation at 4°C	Manually with Ventana reagents
p-mTOR (ser 2448) clone 49F9	Cell Signaling Technology	Pressure cooker, 3 min	1 : 100	30 min at 37°C	Ventana NexES autostainer
mTOR	Cell Signaling Technology	Pressure cooker, 4 min	1 : 50	Overnight incubation at 4°C	Manually with Ventana reagents
Phospho-p70 S6K1 (Thr 389)	Cell Signaling Technology	Pressure cooker, 4 min	1 : 50	Overnight incubation at 4°C	Manually with Ventana reagents
S6K	Abcam	Pressure cooker 6 min	1 : 100	Overnight incubation at 4°C	Manually with Ventana reagents
p-S6RP (ser235/236) clone 91B2	Cell Signaling Technology	Pressure cooker, 3 min	1 : 50	30 min at 37°C	Ventana NexES autostainer
S6RP	Cell Signaling Technology	Pressure cooker, 2 min	1 : 100	30 min at 37°C	Ventana NexES autostainer
p-4EBP1 (Thr70)	Cell Signaling Technology	Pressure cooker, 2 min	1 : 50	Overnight incubation at 4°C	Manually with Ventana reagents
eIF-4E	Cell Signaling Technology	Pressure cooker, 2 min	1 : 50	Overnight incubation at 4°C	Manually with Ventana reagents

**Table 2:** Immunohistochemistry for the AKT/TSC/mTOR pathway molecules in chordomas

	**PTEN**	**CDKN2A**	**p-AKT (ser473)**	**TSC1**	**TSC2**	**p-TSC2 (Thr1462)**	**p-mTOR (ser2448)**	**mTOR**	**p-S6K (Thr389)**	**S6K**	**p-RPS6 (ser235/236)**	**RPS6**	**p-4EBP1 (Thr70)**	**eiF-4E**
0 (no immunoreactivity)	7	46	4	26	0	2	35	11	18	0	41	23	2	1
Low immunoreactivity	28	2	31	14	40	47	5	33	11	50	8	21	25	35
High immunoreactivity	9	0	14			0	8	0	18	0	3	1	21	12
Positive cases/total no.	37/43	2/48	45/49	14/40	40/40	47/49	13/48	33/44	29/47	50/50	11/49	22/45	46/48	47/48
%	86	4	92	35	100	96	27	75	62	100	22	49	96	98

Although the TMA was built with 50 chordomas, the total number described for each antibody represents the number that was possible to analyse.

The scoring system employed was as follows: negative in the absence of immunoreactivity, ‘low’ when the immunoreactivity was unequivocal but less strong than the positive control and ‘high’ when the immunoreactivity was at least as strong as the positive control. In all of the positive cases, the immunoreactivity was present in more than 95% of the neoplastic cells.
