# Supplementary material for: Potential therapeutic targets for chordoma: PI3K/AKT/TSC1/TSC2/mTOR pathway
Source: Br J Cancer. 2009 Apr 28;100(9):1406–14. doi: 10.1038/sj.bjc.6605019 (PMC2694420; doi:10.1038/sj.bjc.6605019)
Supplement: Supplementary Table 1 [file 6605019x1.doc]

Supplementary Table 1: Primer sequences used for mutation analysis of *RHEB and PI3KCA* and RT-PCR for *PTEN*.

| *RHEB* primers | | | |
| --- | --- | --- | --- |
| Gene (exon) | Primer sequence | | Length bp |
| RHEB (exon 2) | F  R | 5’-TTTGTTGAAGGCCAATTTGTG-3’  5’-TGGAGTATGTCTGAGGAAAGATAGAA-3’ | 180 |
| RHEB (exon 4) | F  R | 5’-GGCCAATTTGTGGACTCCTA -3’  5’-TCCCCACCATATCCAACAAT-3’ | 220 |
| *PI3KCA* primers | | | |
| PI3KCA (exon 4) | F  R | 5’-CGCCCCCTTAATCTCTTACA- 3’  5’-TGGATGTTCTCCTAACCATCTG- 3’ | 386 |
| PI3KCA (exon 5) | F  R | 5’-GGCAGCAACTAATTTTGGTGA -3’  5’-ACTTTTTGTAGAAATGGGGTCT- 3’ | 392 |
| PI3KCA (exon 6) | F  R | 5’-TTTCCAATCAATCTCTTTCCTG- 3’  5’-TCCCAAGGTATTCTTCCATGAT-3’ | 396 |
| PI3KCA (exon 9) | F  R | 5’-GCTTTTTCTGTAAATCATCTGTG -3’  5’-CATGCTGAGATCAGCCAAATTC- 3’ | 286 |
| PI3KCA (exon 20) | F  R | 5’-GTCTACGAAAGCCTCTCTAATTT- 3’  5’-TAATGCTGTTCATGGATTGTGC- 3’ | 483 |
| *PTEN* and *GAPDH* primers for RT-PCR | | | |
| PTEN | F  R | 5’-GCGTGCAGATAATGACAAGG-3’  5’-GGATTTGACGGCTCCTCTAC-3’ | 126 |
| GAPDH | F  R | 5’-GATCATCAGCAATGCCTCCT-3’  5’-TGTGGTCATGAGTCCTTCCA-3’ | 72 |

[[1]](#footnote-2)

1. The primers were designed based on the 3’ end transcribed sequence of the test gene with the primer3 design software ([www.broad.mit.edu/cgi-bin/primer/primer3.cgi/primer3_www.cgi](http://www.broad.mit.edu/cgi-bin/primer/primer3.cgi/primer3_www.cgi)). For RT-PCR The primers were designed for different exons to avoid amplification from gDNA [↑](#footnote-ref-2)
