# Supplementary material for: Potential therapeutic targets for chordoma: PI3K/AKT/TSC1/TSC2/mTOR pathway
Source: Br J Cancer. 2009 Apr 28;100(9):1406–14. doi: 10.1038/sj.bjc.6605019 (PMC2694420; doi:10.1038/sj.bjc.6605019)
Supplement: Supplementary Table 3 [file 6605019x3.doc]

Supplementary Table 3: description of the mutations that were screened for PI3KCA

| Exon | Amino Acid |
| --- | --- |
| 4 | p.N345I |
|  | p.N345K |
| 5 | p.C378R |
| 7 | p.C420R  p.E453Q |
| **9** | p.E542K* |
|  | p.E545A |
|  | p.E545G |
|  | p.E545K* |
|  | p.Q546E |
|  | p.Q546P |
| **20** | p.Y1021N |
|  | p.H1047L |
|  | p.H1047R* |
|  | p.H1047Y |
|  | p.H1048L |
|  | p.G1049R |
|  | p.H1065Y |

* 3 oncogenic mutations known to lead to constitutive phosphorylation of AKT and activation of the target of rapamycin (TOR) kinase (Kang et al., 2005)
